# Identification of Telocytes in the Pancreas of Turtles—A role in Cellular Communication

**DOI:** 10.3390/ijms21062057

**Published:** 2020-03-17

**Authors:** Noor Samad Gandahi, Botao Ding, Yonghong Shi, Xuebing Bai, Jameel Ahmed Gandahi, Waseem Ali Vistro, Qiusheng Chen, Ping Yang

**Affiliations:** 1 MOE Joint International Research Laboratory of Animal Health and Food Safety, College of Veterinary Medicine, Nanjing Agricultural University, Nanjing 210095, China; 2017207040@njau.edu.cn (N.S.G.); 2018107021@njau.edu.cn (B.D.); shiyonghong@shvri.ac.cn (Y.S.); 2019207003@njau.edu.cn (X.B.); 2017207039@njau.edu.cn (W.A.V.); Chenqsh305@njau.edu.cn (Q.C.); 2 Department of Anatomy and Histology, Faculty of Animal Husbandry and Veterinary Science, Sindh Agriculture University Tandojam, Sindh 70060, Pakistan; drgandahi@gmail.com

**Keywords:** telocytes, telopodes, cellular communication, exosomes, pancreas, soft-shelled turtle

## Abstract

The existence of telocytes (TCs) has not yet been established in the pancreases of aquatic reptiles. Here, we report TCs in the exocrine pancreas of *Pelodiscus sinensis* using transmission electron microscope (TEM), immunohistochemistry (IHC), and immunofluorescence (IF) techniques. TCs surrounded the acini and ducts of the connective tissue of the exocrine pancreas and between lobules and gland cells. The cells were located preferably close to the blood vessels, interlobular ducts, and nerve fibers. Ultrastructurally, TCs exhibited small and large bodies with thick and thin portions, podoms, and podomers, and prolongations that form dichotomous branching with hetero-cellular and homo-cellular junctions. The podom (thick) portions showed caveolae, mitochondria, rough endoplasmic reticulum, and vesicles. The nucleus carries heterochromatin and is irregular in shape. The shape of TCs depends on the number of telopodes (Tps) bearing long, short, spindle, triangular, and “beads on a string” shapes with twisted, tortuous prolongations and ramifications. Shed extracellular vesicles and exosomes were found frequently released from projections and Tps within connective tissue in the vicinity of the acini and collagen fibers. IHC and IF results showed CD34+, α-SMA+, and vimentin^+^, long and triangle-shaped TCs, consistent with the TEM findings. The presence of shaded vesicles from TCs might implicate their possible role in immune surveillance, tissue regeneration as well as regulatory functions in the reptilian pancreas.

## 1. Introduction

Telocytes (TCs) are interstitial cells of mesenchymal origin, first reported by Popescu and Faussone-Pellegrini in 2005. They were named interstitial Cajal-like cells (ICLCs) and interstitial cells of cajal (ICCs) based on their morphology, ultrastructural features, immunophenotype, and site of existence. Later on, ICLCs were renamed TCs [1,2,3,4]. These are moniliform and long structured cells, that sometimes form convoluted trajectories. These features are different between TCs and other stromal cells such as fibroblast-like cells or fibrocytes [2,5]. The most common significant features are the presence of extremely long projections called telopodes (Tps), which extend from the cell body. Tps present moniliform aspects with much dilatation, resembling “beads on a string” with thin (podomers) and thick (podoms) segments. Tps can reach exceptional lengths of up to 100 μm and can form an attachment with other cells. The cell body of TCs varies in shape (fusiform, pyriform, or triangular) depending on the number of Tps arising directly from the cell body; the latter measures between 9 to 15 µm in length. Podoms show caveolae, endoplasmic reticulum, and mitochondria, and are also involved in the uptake and release of calcium [3,4]. TCs frequently establish homo-junctions (telopodes to telopodes), as well as hetero-junctions with other structures of neighboring cells (nerve fibers, collagen fibers), to perform numerous functions and to form three-dimensional (3D) networks [1,6,7,8,9,10]. 

The distinct morphological features of TCs entail the unique physiological roles in the tissues. More roles of TCs could be expected in the cellular organization of the tissues during development and in the postnatal life. In heart, TCs guide and maintain the tissue organization and complex three-dimensional morphology during development [11], and form mesh-like networks surrounding the hair follicles and sweat glands of skin, guiding the repair of the hair follicles [12]. Similar connective associations were reported in TCs in human exocrine pancreas [13]. Interestingly, in the apolipoprotein-E (ApoE-/-) mice, the animal models for high fat-diet induced atherosclerosis in human arteries, the presence of TCs has been correlated to angiogenesis, repair and homeostasis in the heart, liver and kidney [14,15]. 

Furthermore, TCs secrete extracellular vesicles that play an important role in supporting tissue repair/regeneration and nurse function, intercellular signaling, cell differentiation [16], homeostasis maintenance, and transport and release of lipids, proteins, as well as nucleic acids, in order to meet the target tissue requirements [17,18,19,20,21]. This communication is achieved by releasing the microRNA-filled extracellular vesicles from TCs/Tps to regulate cell functions [22]. Although protein gene product 9·5 (PGP9.5) is a specific tissue marker for the neuroendocrine system and for certain tumors of the lung, colon, and pancreas, a study revealed that CD34, vimentin, tyrosine kinase receptor (c-kit), and PGP 9.5 can be used for TC identification [4]. TCs have been found in the interstitial spaces of the following major organs; namely, intestine [23], lungs [17,24], heart [25], trachea [24,26], parotid glands [27], pulmonary veins [28], pleura [17], skeletal muscle [29], pericardium [25], exocrine pancreas [13,19,30], mesentery [31], gall bladder, placenta [7,32,33], mammary gland, blood vessels [34,35,36,37], uterus, fallopian tube, female reproductive duct [38,39], endometrium [40], and myometrium [41,42]. TCs have been identified in various mammalian species, including humans, pigs, canines, rats, gerbils, mice, and degus [1,6,43,44]. However, there are few studies regarding the characterization of TCs in lower animals like zebrafish, chickens, turtles, and newts [5,19,45]. Extracellular vesicles (EVs) or shade vesicles are nano-sized membrane-bound vesicles that originate in the endosomal compartment. They are shaded from the plasma membrane, and are classified according to their size: (a) exosomes; (b) shading microvesicles or ectosomes; and (c) apoptotic bodies [46]. Antigen-presenting cells release exosomes to induce antigen-specific T cell activation, which is used for immunotherapy. Various cells release ectosomes, used as anti-inflammatory / immunosuppressive agents [21,47]. Innate immunity is an evolutionarily conserved immune system that works against invading pathogens in the front line defense of the host [48]. Cross-link between TCs and other cells influence various complex processes directly or indirectly via gap junctions, through releasing extracellular vesicles, multivesicular bodies, and exosomes [49,50]. TCs are well known for their role in the transmission of intercellular information, homeostasis, the nervous system, stem cell activity, and as immunomodulator cells [51,52].

Chinese soft-shelled turtles (*Pelodiscus sinensis*) are a fresh water species that belongs to the family Trionychidae. The turtle are ectothermic animals, and these animals are, commercially, the most important species of aquaculture in Asian countries. *P. sinensis* have high nutritional and pharmacological values [53], and provide evolutionary-specific links between ectotherms and endotherms, such as fishes, birds, mammals, and amphibians [54]. Therefore, soft-shelled turtles have been used in the field of research as a model animal, in order to provide analysis of the characteristics and functional genes involved in various biological and stress processes [55,56,57]. Furthermore, the pancreas is a crucial vital organ, and ultrastructural and immunophenotyping studies for localization of TCs in the turtle pancreas might suggest the role of these cells in multiple ways, including regeneration/repair, as carriers of information to the neighboring cells, and immune functions [19]. TCs share organ specific characteristics among different species [5,13], similar functions of TCs may be expected in mammals.

Our present study confirms the presence of TCs within the connective tissue in the lobules and gland cells within the pancreas of Chinese soft-shelled turtles (*Pelodiscus sinensis*) via transmission electron microscope (TEM), immunohistochemistry (IHC), and immunofluorescence (IF). Close associations between TCs and other neighboring cells are reported in blood vessels (BVs), smooth muscle cells (SMCs), nerve fibers (NFs), nerve tracts, inter lobular ducts, gland cells, and collagen fibers (CFs). Extracellular / shed vesicles from podoms and Tps in the vicinity of BVs, CFs, and ducts were observed. This will help for understanding the role of the pancreas in innate immune functions, tissue regeneration in the field of cell biology, and ultrastructural insights into the possible functions of TCs in reptiles. 

## 2. Results

### 2.1. General Structure of the Pancreas

The pancreatic tissue chiefly consists of glandular lobules composed of acinus, islets, and connective tissue separating these lobules. Within the lobules, lots of small blood vessels surround the glands, but only few telocytes can be found. However, it is difficult to distinguish between telocytes and endothelial cells in IHC and IF, although TEM is good for their identification. A lot of TCs exists in the connective tissue, large blood vessels, and ducts separating these lobules. Secretory ducts are either single cuboidal epithelium or single columnar epithelium. However, vascular endothelium is a single layer of flattened epithelium.

### 2.2. TEM Analysis of Telocytes in the Pancreas of Chinese Soft-Shelled Turtles

**Figure 1:**
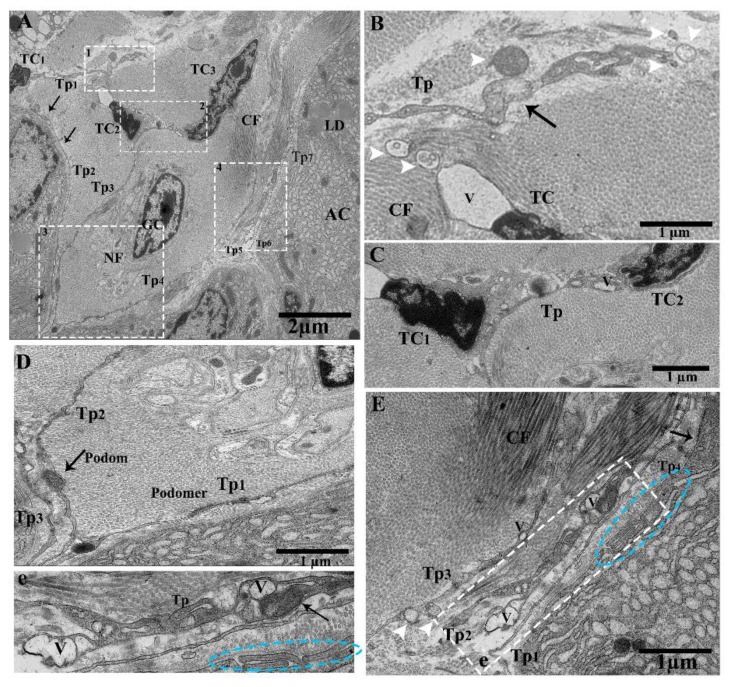
Ultrastructure of telocytes between lobules in connective tissue, shading vesicles/extracellular vesicles releasing form podoms, close connection with Tps–Tps and collagen fiber. Transmission electron microscope (TEM) images of a turtle pancreas showing close proximity with nerve cells, glial cells, the acinar, and collagen fibers. (**A**) Contains three TCs (TC_1_, TC_2_, TC_3_). The nucleus contains heterochromatin and a peripheral attachment with an irregular shape. The cell body of TC_2_ and TC_3_ bearing junctions at TP_3_. Long beaded-like telopodes, Tps (Tp_2_, Tp_3_, Tp_4_, Tp_5_, and Tp_6_), harboring alternating podoms and podomers (arrows). The square-marked areas 1, 2, 3, and 4 in (**A**) are enlarged in (**B**,**C**,**D**,**E**), respectively. (**B**) Extracellular vesicles (EVs; white arrowheads) released from the podoms in the connective tissue in the vicinity of collagen fibers (arrow). (**C**) TC_1_ and TC_2_ have a common connection—their Tps contain small bubble-like vesicles. (**D**) Long cylinder Tps (Tp_1_, Tp_2_, Tp_3_), which have common nano-connections with thick and thin segments (podoms and podomers) and contain mitochondria and vesicles in their podoms. (**E**) Enlarged in (**e**). The Tps contain homo-cellular and hetero-cellular junctions between Tps–Tps and collagen fibers, and contain mitochondria, caveolae, and vesicles in their podoms (arrow). Blue circled area in (**E**,**e**) shows the short overlap of Tps, and the white arrowheads indicate EVs. TCs, telocytes; Tps, telopodes; GC, glial cells; NF, nerve fibers; rER, rough endoplasmic reticulum; podoms and podomers, arrows; LD, lipid droplet; AC, acinus; CF, collagen fibers; V, vesicle. Scale bar = 2 µm in (**A**); scale bar = 1 µm in (**B**–**E**).

TEM images showed pancreatic telocytes; these cells were observed in close proximity to the acinus, collagen fibers, large blood vessels, gland cells, nerve tracts, ducts, and glial cells, comprising long and short prolongations of telopodes. They were also shown to contain typical podoms, as well as podomers that were easy to recognize. The nucleus was observed to contain heterochromatin in clusters and to be irregular in shape, attached with the peripheral nuclear envelope (Figure 1A). Numerous extracellular/shed vesicles were released from the podoms at the end point of the telopodes in the connective tissue in close proximity to collagen fibers (Figure 1B). We observed that TC_1_ and TC_2_ were contiguous (Figure 1C); the close contacts of the telopodes were with neighboring acini, nerve, and glial cells. Each of them—Tp_1_, Tp_2_, and Tp_3_—had long spindle-shaped Tps, the podoms contained mitochondria and caveolae, and the podomers had homo-junctions between Tp_1_, Tp_2_, and Tp_3_ (Figure 1D). Several pinching-off/shed vesicles were observed (Tp_2_, Tp_3_), along with collagen fibers (Figure 1E). Vesicles and overlapped Tps were also observed (Figure 1e). The releasing of extracellular vesicles/shed vesicles suggests homo-cellular communications, as well as the exchange of intracellular material between neighboring cells, Tps, and TCs through secretory vesicles in the connective tissue (Figure 1B,E).

**Figure 2:**
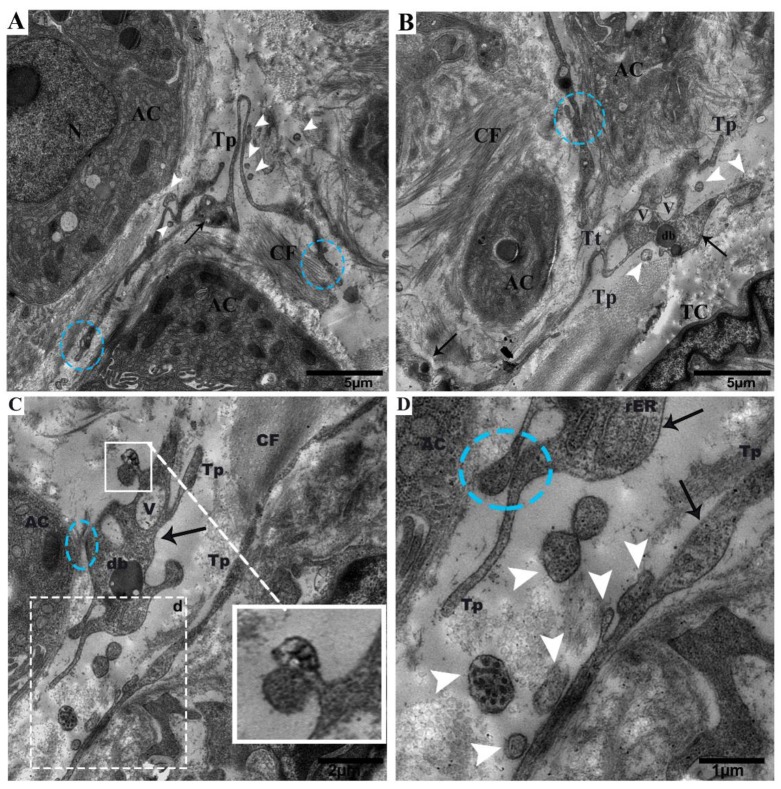
Tps within the connective tissue budding and shedding microvesicles and rER in podoms, in between gland cells, making close connections with the centroacinus and neighboring cells. (**A**) Long rope-like Tps with a convoluted process forming junctions and close connections with collagen fibers (blue circle). The podoms have mitochondria and vesicles (black arrow). Shed vesicles within connective tissue (white arrowheads). (**B**) TCs have heterochromatin peripheral nuclei. Tps form a tortuous process in close proximity to the centroacinus, as well as close connections with gland cells and collagen fibers (blue circle). The podom contains vesicles and dense bodies (black arrows). Shed microvesicles from the podomic area within the connective tissue. Tps form a long process. (**C**) Vesicles and dense bodies (black arrow), and a close connection with the acinus (blue circle). A magnified view of budding extra cellular vesicles from the podom (white square). White circled area d in (**C**) inset into (**D**), respectively; in the podom rough endoplasmic reticulum (black arrow), close connections with podoms to podoms and the acinus can be seen (blue circle). Numerous shedding microvesicles within the connective tissue (white arrowheads) can be seen in (**D**). TC, telocyte; CF, collagen fibers; Tp, telopodes; Tt, tortuous process; db, dense body; V, vesicles; AC, acinus. Scale bar = 5 µm in (**A**,**B**); scale bar 2 µm in (**C**); scale bar = 1 µm in (**D**).

Tps were located within the connective tissue close to the centroacinar and surrounding cells (collagen fibers and acinus) (Figure 2A,B). Tps shed microvesicles within the connective tissue and formed a long rope-like convoluted process (Figure 2A), a tortuous process, and vesicles (Figure 2B). The podoms were also shown to contain rough endoplasmic reticulum (rER)(Figure 2C), and the Tps and podoms shed numerous microvesicles in the vicinity of and in close connection with the acinus (Figure 2C,D). Budding extracellular vesicles from the podoms were also observed (Figure 2C).

**Figure 3:**
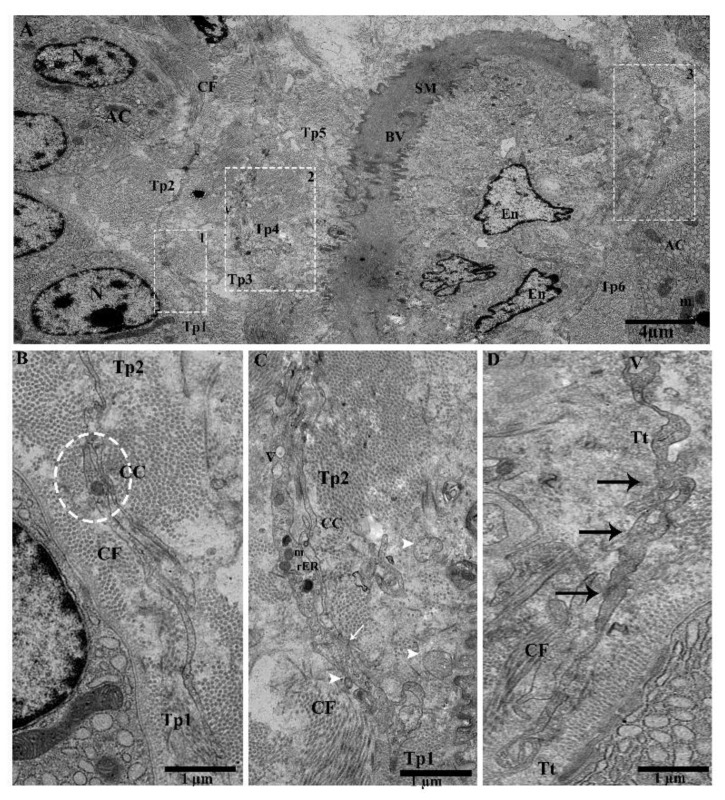
The connective tissue in between the lobules contains Tps around the large blood vessels, releasing extracellular vesicles and their tortuous processes, and beads on a string and close connections around the bend with blood vessels and the acinus can be observed. (**A**). Tps in the vicinity of blood vessels and adjacent to the acinus, scattered around and showing large prolongation. Square-marked areas 1, 2, and 3 in (**A**) are enlarged in (**B**,**C**,**D**), respectively. (**B**) Long cell process (Tp_1_, Tp_2_), and close connections (junctions) between podoms to podomers and adjacent to the acinus. (**C**) In the vicinity of a dilated segment of Tp_1_, rough endoplasmic reticulum, mitochondria, dense bodies, vesicles, and close connections (podoms–podomers) with Tp_1_ (white arrow) are visible. EVs (arrowheads) are present in connective tissue. (**D**) Tps contain a tortuous process, with many twists (beaded-like) (black arrows), along with collagen fibers and the acinus. Tp, telopodes; BV, blood vessel; SM, smooth muscle cells; En, endothelial cells; AC, acinus; CF, collagen fibers; V, vesicles; CC, close connection; Tt, tortuous; m, mitochondria; rER, rough endoplasmic reticulum; db, dense body. Scale bar = 4 µm in (A); scale bar = 1 µm in (**B**–**D**).

We observed Tps within the connective tissue between the lobules around large blood vessels, along with the acinus and collagen fibers. Tps have long dilatations forming a close connection, podoms to podoms, and dichotomous branching (Figure 3A). A close connection can be observed between Tp_1_ and Tp_2_ (podoms to podomers), adjacent to the acinus (Figure 3B). Furthermore, numerous shed / secretary vesicles in the vicinity of blood vessels can be seen in the connective tissue, where the thick portion contains dense bodies and vesicles (Tp_1_), in close contact with podoms to podomers (Tp_2_) (Figure 3C). Tps contain the winding way (beads on a string) and the tortuous process (Figure 3D). It is observed that in large blood vessels, the functions of Tps secretary vesicles in connective tissue include to repair and protect against destruction or obstructions of vessels through chemical secretions, to enhance the peristalsis functions of smooth muscle cells and endothelial cells, and to control the passage of materials, as well as white blood cell transmission in and out of the blood stream.

**Figure 4:**
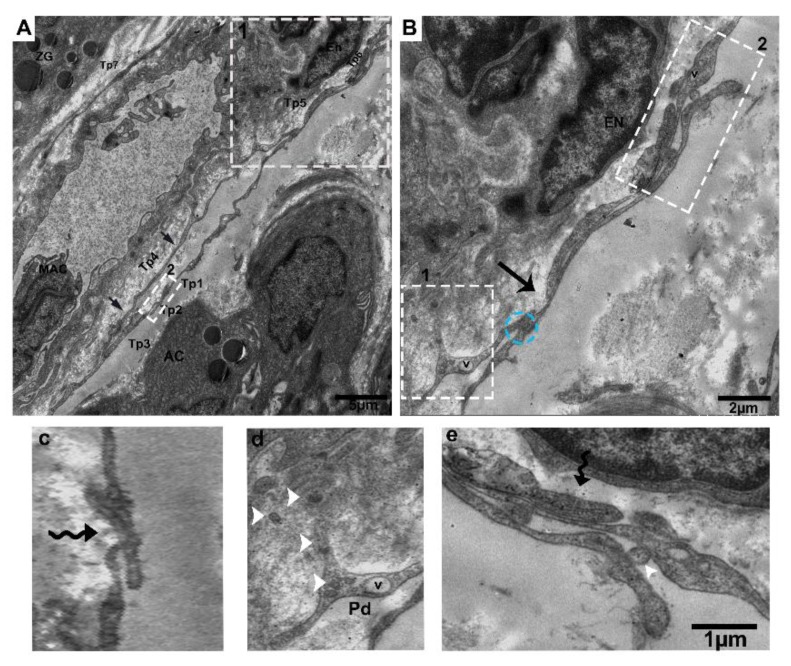
Tps inside and surrounding the interlobular duct with different patterns of long and short overlapping podoms, and podomers within the connective tissue between the acinus. (**A**) Tps forming a long, convoluted process with many overlapping twists (black arrowheads), and the inside of the interlobular duct has podoms with vesicles. Square-marked areas in (**A**) inset into (**B**,**C**), respectively; (**B**) short overlap and punctate contact (blue circle) and gap junctions, and short overlap (black arrow). (**c**) Hook joint and short overlap with tight junctions between the three Tps. Square-marked areas in (**B**) inset into (**d**,**e**), respectively; (**d**) shed/extracellular vesicles within the space of the interlobular duct from the podom (white arrowheads). (**e**) A short overlap between podoms to podoms and podomers and shed vesicles (white arrowheads), in closeness to the endothelial cells. Tp, telopodes; MAC, macrophage; AC, acinus; E, endothelial; ZG, zymogen granules; V, vesicles; Pd, podoms. Scale bar = 5 µm in (**A**); scale bar = 2 µm in (**B**); scale bar = 1 µm in (**e**).

Tps surround the interlobular duct long and short convoluted processes with many twists, with overlapping of the podoms and podomers between the acinus, where the macrophage is shown inside the duct surrounded by Tps (Figure 4A,B). The following can also be observed: Short overlap and punctate contact (Figure 4B); short overlap with hook joint, forming tight junctions between Tps (Figure 4C); numerous shedding microvesicles shed within the interlobular duct and the endothelial cell space (Figure 4D); and overlapping between podoms to podoms, as well as closeness with endothelial cells (Figure 4E).

**Figure 5:**
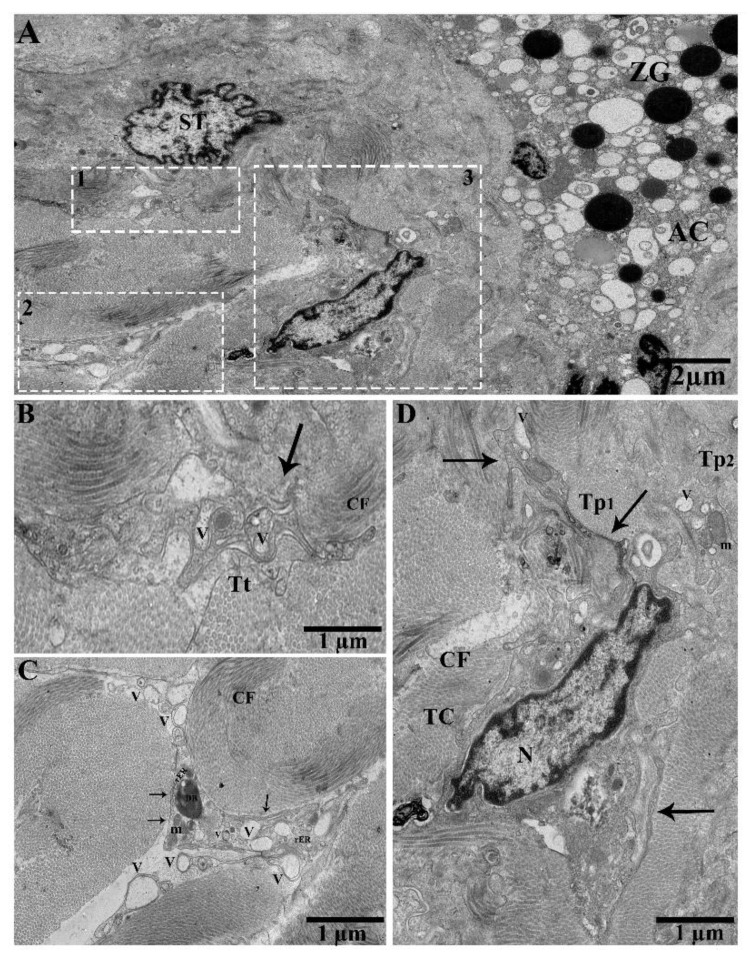
In between gland cells, small blood capillaries, and numerous shed/extracellular vesicles from the podoms in the connective tissue in the vicinity of collagen fibers. (**A**) TEM photograph in the pancreas showing the acinar cells, zymogen granules, and stellate cells. Square-marked areas 1, 2, and 3 in (**A**) are enlarged in (**B**,**C**,**D**), respectively. (**B**) Tortuous process and vesicles (black arrow). (**C**) Podoms containing numerous extracellular/shed vesicles, scattered in the vicinity of collagen fibers, and rough endoplasmic reticulum, mitochondria, and dense bodies are present. (**D**) A typical TC, which contains heterochromatin in their periphery, forming Tp_1_ vesicles (arrows). Tp_2_ contains mitochondria and vesicles in the podoms. N, nucleus; ZG, zymogen granules; AC, acinus; ST, stellate cell; Tt, tortuous process; V, vesicles; m, mitochondria; rER, rough endoplasmic reticulum; DB, dense body; CF, collagen fibers. Scale bar = 2 µm in (**A**); scale bar =1 µm in (**B**–**D**).

We observed TC in between gland cells, along with small blood capillaries, stellate cells, and collagen fibers. The functions of these secretions with stellate cells may be suggestive of multiple functions in tissue repair/regeneration, as well as of pathological conditions (Figure 5A). Tp also showed tortuous processes (Figure 5B). Podoms contain numerous vesicles, dense bodies, mitochondria, rough endoplasmic reticulum, and extracellular/shed vesicles in the connective tissue in the vicinity of collagen fibers (Figure 5C). TC contains heterochromatin in the periphery, a long Tp process with vesicles (Tp_1_), and Tp_2_ mitochondria and vesicles (Figure 5D).

**Figure 6:**
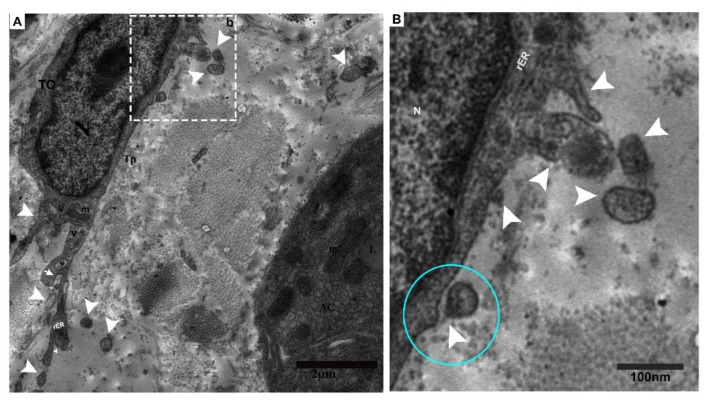
Pinching off vesicles and numerous shading vesicles within the connective tissue. (**A**) The nucleus of TC contains central euchromatin and peripheral heterochromatin, and Tps have visible mitochondria and vesicles. Coated pit releasing shed vesicles (white arrow), as well as multiple releasing and shade vesicles within the connective tissue (white arrowheads), can be seen. The square-marked area (**b**) in (**A**) is inset into (**B**) pinching off vesicle (blue circle). The podoms contain rough endoplasmic reticulum and mitochondria, as well as releasing shade/extracellular vesicles (white arrowheads). N, nucleus; TC, telocyte; Tp, telopodes; V, vesicles; m, mitochondria; rER, rough endoplasmic reticulum; LD, lipid droplet; AC, acinus. Scale bar = 2 µm in (**A**); scale bar = 1 µm in (**B**).

Furthermore, TCs pinch off and shade vesicles from the cytoplasm. Coated pit releasing shade microvesicles can be seen, as well as podoms that contain rER and vesicles (Figure 6A,B).

**Figure 7:**
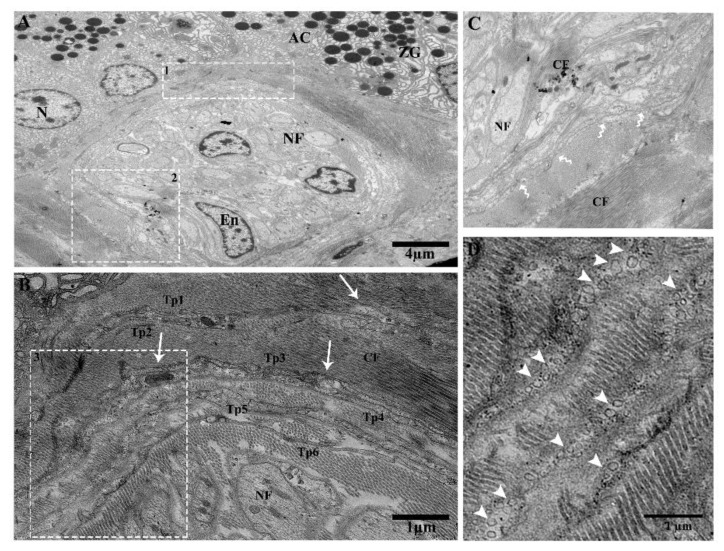
Large duct attached with lobules through the connective tissue in between the gland cells. Tps surround the ductus and form triangular structures and shading vesicles in the connective tissue. (**A**) Telopodes establishing a triangular structure “trio” adjacent to the acinar cells and collagen fibers, nerve fibers, and telopodes showing nano-contacts. Square-marked areas 1 and 2 in (**A**) are enlarged in (**B**,**C**) respectively. (**B**) Tp_1_, Tp_2_, Tp_3_, and Tp_4_ have gap junctions, and there are close connections between Tp_5_ and Tp_6_. The podoms contain mitochondria and caveolae (white arrows). (**C**) In the vicinity of collagen fibers, nerve fiber Tps junctions (podoms–podoms) and segments (bent arrows) can be seen. The square-marked area 3 in (**B**) is enlarged in (**D**), where numerous shading vesicles (arrowheads) in the connective tissue, along with collagen fibers, at the ending point of Tp_1_, Tp_2_, Tp_3_, Tp_4_, Tp_5_, and Tp_6_ are often captured, which suggests a transfer of information between telopodes and adjacent cells. Tp, telopodes; CF, collagen fibers; NF, nerve fiber; En, endothelial cells; AC, acinus; ZG, zymogen granules. Scale bar =4 µm in (**A**); scale bar = 1 µm in (**B**,**D**).

The lobular duct contains Tps in the connective tissue surroundings and forms a triangular-shaped “triad” in clusters with collagen fibers in between the gland cells (Figure 7A). The telopodes are parallel to each other and form junctions, containing mitochondria and caveolae (Figure 7B). Overlapping among Tps was observed (Figure 7C). Multiplex shed/secretory vesicles can be seen within the connective tissue and collagen fibers that exchange molecules with neighboring cells and gland cells (Figure 7D).

**Figure 8:**
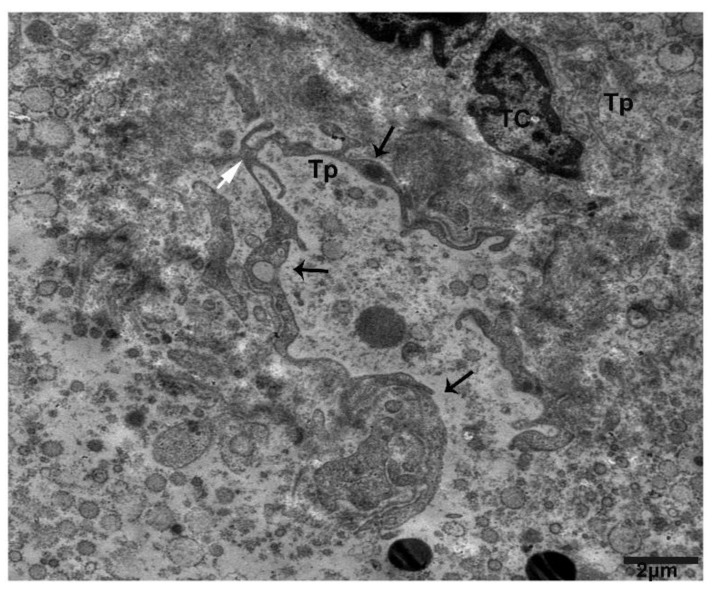
Numerous clusters of shade/extracellular vesicles from Tps within the connective tissue between lobules. The TC nuclei bears heterochromatin in their periphery. Within the connective tissue, there are multiplex clusters of shading/extracellular vesicles scattered around, and the podoms contain vesicle and mitochondria (black arrows), forming trajectories from the podomic area (white arrow). Scale bar = 2 µm.

Tps form a trajectory and convoluted process, releasing/shedding numerous clustered and scattered extracellular vesicles from the podoms within connective tissue (Figure 8).

### 2.3. IHC Analysis of Telocytes in the Pancreas of Chinese Soft-Shelled Turtles

**Figure 9:**
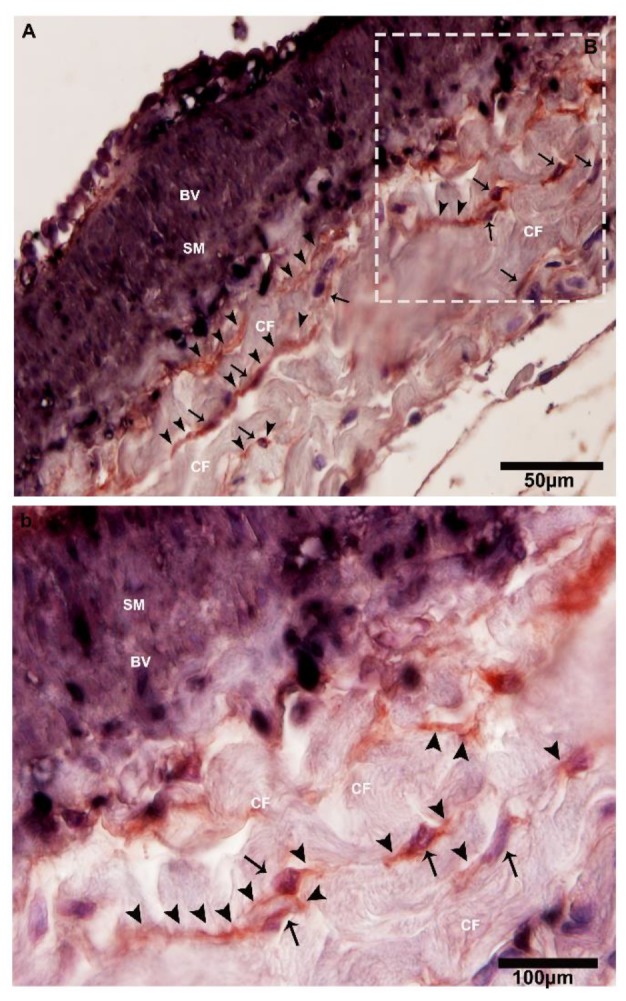
CD34-positive and α-SMA-positive telocytes around the large blood vessels in the connective tissue. (**A**) The square-marked area B in (**A**) is enlarged in (**b**). Immunohistochemistry (IHC) analysis shows at lower magnifications the CD34-positive and α-SMA-positive cells adjacent to large blood vessels. At high magnifications, CD34 staining has strong positivity with dilated portions, whereas α-SMA stains positive for smooth muscle layers and blood vessels. Higher and lower magnifications of IHC (two anti-bodies—CD34 and α-SMA) were used. The square-marked area B in (**A**) is enlarged in (**b**). The telocytes and their nuclei can be seen (black arrows), showing long cellular projection telopodes (black arrows head) and displaying thin and thick (podoms and podomers) segments in the vicinity of collagen fibers and adjacent to the blood vessels. BV, blood vessels; CF, collagen fibers; SM, smooth muscle. Scale bar = 50 µm in (**A**); scale bar = 100 µm in (**B**).

**Figure 10:**
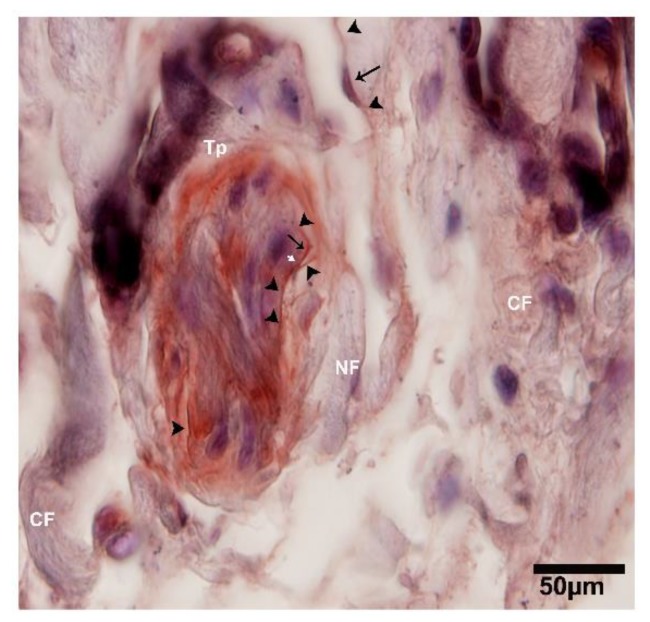
CD34-positive and α-SMA-positive for TCs and endothelial cells, where TCs surround the large duct within the connective tissue. At lower magnification image of IHC stained CD34-positive. Around the duct, Tps show a cluster deposition and form a triangular shape, along with the nerve fibers. The nuclei of the telocytes are stained blue and the podoms are indicated with the black arrows, while the prolongations of cells are shown with arrowheads and the junctions between two Tps are indicated with the white arrow. NF, nerve fibers; CF, collagen fibers; Tp, telopodes. Scale bar = 50 µm.

IHC stains CD34-positive and α-SMA-positive blood vessels and nerves at lower magnifications (Figure 9A,B and Figure 10). However, CD34-positive staining shows dilated segmentation (podoms) and strong positivity at higher magnification (Figure 9A and Figure 10). Next, a uniform α-SMA positive staining is observed through the smooth muscle layers and blood vessels (blue black) (Figure 9A,B).

### 2.4. Double IF Analysis in the Pancreas of Chinese Soft-Shelled Turtles

**Figure 11:**
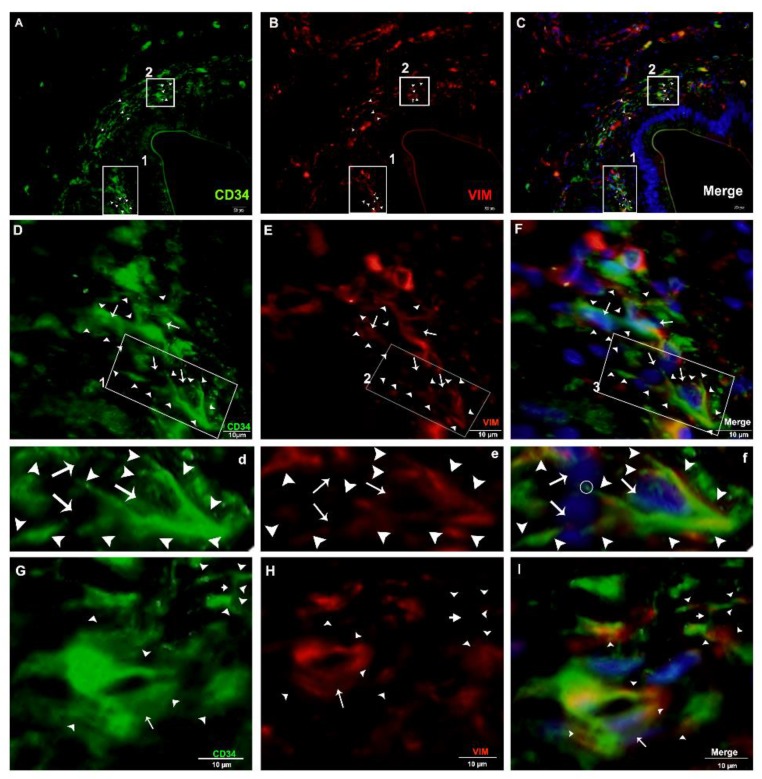
Immunofluorescence-positive TCs in the connective tissue around the interlobular ducts (CD34+, Vimentin+, and merged). (**A**–**C**) CD34+ (green), Vimentin+ (red), and merged of both showed telocytes and their prolongations abundantly scattered around ducts (arrowheads), respectively. The square-marked area 1 in (**A**,**B**,**C**) is enlarged in (**D**,**E**,**F**), respectively, and the square-marked areas in (**D**,**E**,**F**) are enlarged in (**d**,**e**,**f**), respectively. Furthermore, a spindle-shaped (cone-shaped) TC with a nucleus (arrows) and two long process (trajectories) connected with other TC_s_ with their prolongations (arrowheads) can be observed. The square-marked area 2 in (**A**,**B**,**C**) is enlarged in (**G**,**H**,**I**), each with a triangular-shaped TC with one process. In addition, two Tps with long processes (right-sided) connected by a junction (small arrows), a nucleus (arrows), cell processes (arrowheads), and junctions (small arrows) can also be seen. Scale bar = 20 µm in (**A**–**C**); scale bar = 10 µm in (**D**–**F**); scale bar = 10 µm in (**G**–**I**).

**Figure 12:**
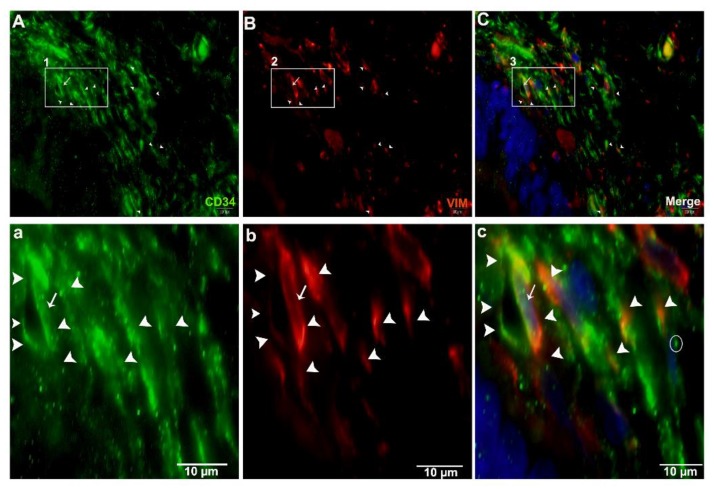
Immunofluorescence-positive telocytes within the connective tissue around the lobular duct. Immunofluorescence CD34+ is shown in green, and Vimentin+ in red. Telocytes were scattered around the lobular duct. The square-marked areas 1, 2, and 3 in (**A**,**B**,**C**) are enlarged in (**a**,**b**,**c**), respectively, with a triangle TC with a long process near the other telopodes, as well as a nucleus (white arrow) and Tps (arrowheads). Scale bar = 10 µm in (**A**–**C**); scale bar = 10 µm in (**a**–**c**).

**Figure 13:**
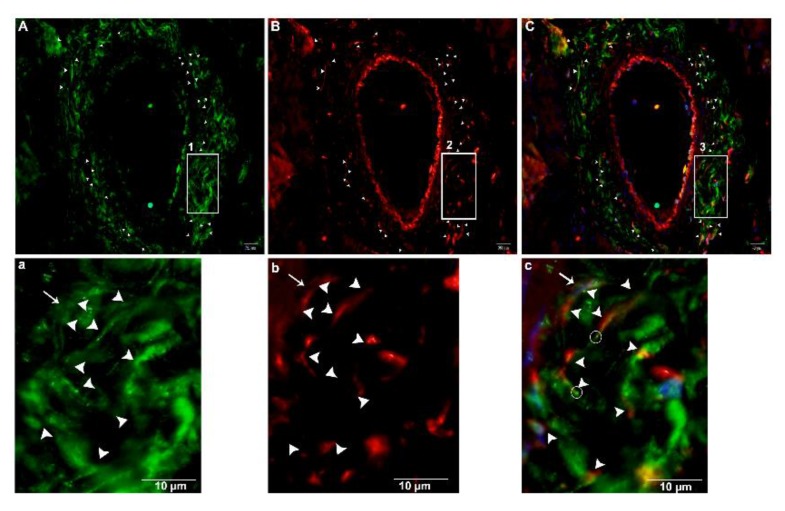
Immunofluorescence-positive telocytes in the connective tissue surrounding large blood vessels. Immunofluorescence CD34+ is shown in green in (**A**), Vimentin+ in red in (**B**), and merged in (**C**), and labeled. Tps were abundantly distributed around the large blood vessels (arrowheads). The square-marked areas 1, 2, and 3 in (**A**,**B**,**C**) are enlarged in (**a**,**b**,**c**), respectively. TCs with Tps and long processes close to the other telopodes can be seen. (**c**) Podom, marked as a bright spot (circle), the nucleus (arrow), and the processes (arrowheads) are all visible. Scale bar = 20 µm in (**A**–**C**); scale bar = 20 µm in (**a**–**c**).

For the justification of both CD34+ and Vimentin+, we performed double immunofluorescence. Vimentin was positive for cytoplasm, and CD34 for cytomembrane. These positive cells were TCs (Figure 11A–C). In the interlobular ducts, the TCs in the connective tissue were distributed in clusters and interwoven, forming a network with the neighboring cells (Figure 11D,F) and prolongations with dichotomous branching that bears spindle-shaped or cone-shaped cells with nucleus, as indicated by a bright spot (podom) (Figure 11d,e). These cells were shown to be triangle structures and formed junctions between the Tps (Figure 11G,I). At a high magnification, TCs and Tps were scattered in the connective tissue around the lobular duct, and stained CD34+ and Vimentin+. These cells were interwoven, forming a triangle shape with a visible nucleus (Figure 12A(a),C(c)). Vimentin stained positive for the walls of blood vessels, and CD34 (red) positive for the surrounding cells, endothelial cells, and blood vessels. In the connective tissue, TCs in stained in little quantity with nuclei, and form long cell process., Tps that are abundantly distributed around the blood vessels form intertwined, dichotomous branching with neighboring cells, with bright spots indicating the long process of the Tps podoms (Figure 13A(a),C(c)).

Consistent with the TEM analysis, all cells appeared to be CD34+, and all cells were confirmed to be TCs.

## 3. Discussion

This is the first study to identify TCs in the pancreas of Chinese soft-shelled turtles. We demonstrated the morphological and ultrastructural characteristics of these cells by TEM, IHC, and double IF. TEM is the best way for accurate identification of TCs [1,2,29]. We know that TCs are a subpopulation of interstitial mesenchymal cells, and the importance of TCs during the mesenchymal embryonic stages of the pancreas is well known [58,59]. They establish intercellular communications between Tps and other neighboring cells by small signal molecules (e.g., proteins, RNAs, and microRNAs) through some secretions, shedding/extracellular vesicles, micro vesicles, ectosomes, and exosomes within the extracellular matrix. Furthermore, the occurrence of this function in the myocardium has also been proposed [1,60,61]. Previous work shows the extracellular vesicles communicating in TCs mediated by intercellular communications [4,17,61]. In the present study, it was shown that TCs have intercellular communications with nearby cells through shading/extracellular vesicles. It has been suggested that TCs can release EVs within different spaces of stromal organs [17,60,61]. Through studies, it has been revealed that EVs are frequently derived from projections, but rarely in the culture from the body of TCs [1,60,62]. However, the extracellular/shading vesicles in this study were frequently observed and shade from projections, podoms, and pinching-off from cell cytoplasm and telopodes in the gland cells and in the vicinity of collagen fibers, interlobular duct, and large blood vessels and capillaries surrounding the nerves. These observations may possibly explain the influence by the site of the sample cutting and the sectioned angles. The culture of cardiac TCs releases different sizes of extracellular vesicles, measuring: multivesicular bodies (1 ± 0.4 µm), exosomes (45 ± 8 nm), and ectosomes (128 ± 28 nm) [62]. In our present work, shade/extracellular vesicles or secretory vesicles were observed in the connective tissue within the intracellular and extracellular spaces, in between telopodes, NFs, collagen fibers, and blood vessels. In the vicinity of telopodes, we found EVs near NFs, CFs, ACs, and blood vessels. These buds originate from the telopode’s plasma membrane. On the basis of the above findings, we hypothesized that the shape of the telopodes is responsible for the vesicles’ secretion and delivery into the extracellular matrix, suggesting intercellular communication between cells to other cells, NFs, CFs, BVs, and ACs, of either short or long distances. TCs, through their podoms, release extracellular vesicles in the case of damaged blood vessels or tissue cell injuries, and emits chemical signals that cause it to synthesize the appropriate proteins to repair the injury. Where the target is smooth muscle cells, podoms create peristaltic contraction frequency to target cells. If the target cell is a macrophage or a lymphocyte, the exosomes transfer specific immune reactions. Telocytes reduce the excessive work, because it supplies exosomes locally and automatically, so as to reduce the expenses of cellular machinery in the dissemination of information over short and long distances [63,64].

TCs have different distinguishing features, short cell processes, and protrusion with podoms (thick) and podomers (thin). Furthermore, Tps are long, convoluted, and moniliform. The podomic “triad” (dilated segment) consisting of caveolae, mitochondria, and endoplasmic reticulum is involved in Ca^2+^ movements [65]. Our results clearly show the existence of long, convoluted, and moniliform Tps. Previous investigation showed that TCs are present in the pancreas of several mammals, cats, rats, degus, and humans [13,19,30,43]. In this study, we demonstrated the presence of TCs within the connective tissue in between the lobules and gland cells in the pancreas of Chinese soft-shelled turtles by TEM, IHC, and IF, according to the TC diagnostic criteria. α-SMA+ immunoreactivity has been shown in the peritubular myoid cells (smooth muscle-like cells) in the wall of seminiferous tubules, while CD34+ staining is reported in podoms of telocytes in seminiferous tubules of rat testis [66]. Some studies claim that some of the CD34+ TCs can also be positive for α-SMA+, serving as the source cells for differentiation into fibroblasts and myofibroblasts. These dual immunophenotype TCs serve in the repair of damaged sites through formation of granulation tissue and fibrosis [67]. There has been a wide debate on TC-specific antigens. TCs from virtually all human tissues reported so far, are consistently positive for CD34 glycoprotein. Thus, they are also referred to as TCs/CD34+ stromal cells [6,8,68]. Consistently, CD34+ TCs are also reported in other mammals, amphibia and reptiles. Previous studies report CD34+ telocytes in human exocrine pancreas [13], in the rumen of goat [4], in the pancreas of Chinese salamander [19], and in the oviduct [5] and testicular parenchyma of Chinese soft-shelled turtle [45]. Using double immunofluorescence analysis in the current study, we were able to exclude the presence of CD34+/α-SMA+ myofibroblast-like telocytes. However, these TCs co-expressed CD34+/Vimentin+ immunoreactivity in the turtle pancreas.

Developmental studies on lungs in rabbit have established the proportion between TCs during gestational and postnatal stages. There was a significant reduction of population and cell diameters of TCs, while similar increment in the length of Tps during the advancement during postnatal life. The study concluded that the TCs contributed to the angiogenesis, establishment of blood-air barrier and organization of the lung parenchyma during the development [69]. TCs and lymphatic endothelial cells (LECs) were reported as immunophenotypically distinct and spatially close cells in human tongue, colon and skin tissues. TCs were CD34+/PDGFRα+/PDPN-/LYVE-1-; conversely, LECs were CD34-/PDGFRα-/PDPN+/LYVE-1+ [70].

Amphibian telocytes have a classical similarity in ultrastructure to mammals. The difference in TCs between amphibians and mammals is that Tps of amphibian TCs are not convoluted like mammalian telopodes, perhaps because they do have dichotomous branching [1]. Previously, it was revealed that the pancreas of amphibia might widely show the existence of TCs, and have different evolutionary status. However, in tissue, the role of telocytes has not been thoroughly determined, but it is believed that TCs control growth, communication, regeneration of neighboring cells and stem/progenitor cells, and tissue repair [29,71,72]. Previous studies have shown that in the pancreas of mammals, TCs are mainly located in the stroma [13,30]. However, some are in between the pancreatic acinar cells [19,73]. There is also a close association of TCs and Tps with capillaries, acinar cells, stellate cells, glial cells, mast cells, nerve fibers, macrophages, and pancreatic ducts [13,30]. On the basis of structural characteristics, the location of TCs to other cell types of could play a role in intercellular signaling [13]. The pancreatic duct functions as a pacemaker for duct motility, and for spontaneous rhythmic pancreatic duct contractions [73]. However, studies have also demonstrated that TCs are often observed as adjacent in acinar cells, ducts, nerves, and blood vessels in the amphibian pancreas [13,19,30,43].

In this study, we demonstrated the presence of TCs and Tps within the connective tissue in between lobules and gland cells. Within the connective tissue, there are a lot of TCs, and their long elongated Tps surround the large blood vessels and the interlobular duct. In between the gland cells, a small amount of Tps and a large quantity of small blood capillaries exist. Moreover, the shed/extracellular vesicles can also be found from the podoms within the connective tissue in between the gland cells, collagen fibers, large and small blood vessels, Tps, and interlobular duct. On the basis of this evidence, it is suggested that TCs and Tps within the connective tissue in between lobules and gland cells in the pancreas of Chinese-soft shelled turtles, along with large and small blood vessels, interlobular ducts, nerve fibers, and collagen fibers, may be functionally related. Accordingly, telocytes may play a functional role in the immune reactions and secretion in the pancreas, the endocrine, as well as the exocrine system.

## 4. Materials and Methods

### 4.1. Animals and Tissue Block Preparation

Ten adult (of either sex) Chinese soft-shelled turtles, *P. sinensis* (3–4 years), were purchased from a wild breeding base in Jiangsu province of China. Each animal weighed 650–750 g. The animals were anesthetized by sodium pentobarbital (20 mg/kg body weight), administered intraperitoneally, and were then killed by cervical dislocation. The pancreas samples were collected for transmission electron microscopy. The experimental procedure was so designed as to minimize the suffering of the animals. The slaughtering and sampling procedures were approved by the Nanjing Agricultural Veterinary Experimental Animal Ethics Committee and Science and Technology Agency of Jiangsu Province (SYXK (SU) 2010-0005, approved on 1 January 2010).

### 4.2. Transmission Electron Microscopy (TEM)

Blocks of turtle pancreas approximately 1 mm^3^ in size were fixed in 2.5% glutaraldehyde in 0.1 MBPS solution (pH 7.4) at 4 °C overnight. Post-fixation of the turtle pancreas was done in 1% osmium tetroxide (Polysciences Inc. Warrington, PA, USA) for 1 h. The specimen washing was done in distilled water and stained en bloc with 2% aqueous uranyl acetate, then washed again in distilled water (3 × 5 min). Dehydration was done in ethanol, and specimens were embedded in Epon 812 R (Merck, Whitehouse Station, NJ). Ultrathin 50 nm-thick sections were mounted on copper grids and contrasted with uranyl acetate and lead acetate. Specimens were observed under the Hitachi TEM system (H-7650, Tokyo, Japan).

### 4.3. Immunohistochemistry (IHC)

The turtle pancreas samples used 10% formalin for tissue fixation. Tissue samples were first embedded and made into paraffin wax blocks, and then tissue sections were cut into 6 µm before being deparaffinized and rehydrated. Ethyl alcohol (100%, 100%, 95%, 95%, 85%, 75%) was then used in decreasing concentrations. Samples were incubated in 3% hydrogen peroxide to block endogenous peroxidase activity for retrieval of antigen. Bovine serum albumin (BSA) 5% was used for blocked specimens, which were placed for 30 min at room temperature, followed by incubation with rabbit anti-CD34 (1:100 dilutions; catalog no BA3414; Boster Wuhan, China) and mouse anti-αSMA (1:100 dilutions; catalog no. BM0002; Boster, Wuhan China) antibodies for 24 h at 4 °C. Sections were washed and incubated using biotinylated anti-rabbit/anti-mouse IgG (Boster Bio-Technology, Wuhan, China), then placed for 1 h at room temperature. The sections were incubated with avidin-biotinylated peroxidase complex. Next, the activity of peroxidase was determined by staining with diaminobenzidine (DAB, Boster Bio-Technology, Wuhan, China), and hematoxylin stain was used for the nucleus.

### 4.4. Double Immunofluorescence (IF)

The paraffin sections of the pancreas tissue were deparaffinized, followed by antigen unmasking in sodium citrate buffer. Then, for block non-specific antibody binding, the tissue sections were incubated with 1% BSA for 30 min at room temperature. Following this, the samples were incubated overnight at 4 °C, with primary antibody pairs: mouse anti-vimentin (1:100 dilutions; catalog no BA0135; Boster Bio-Technology, Wuhan, China), rabbit anti-CD34 (1:100 dilutions; catalog no BA3414; Boster Bio-Technology, Wuhan, China), and mouse anti-αSMA (1:100 dilutions; catalog no. BM0002; Boster, Wuhan, China). Then, after washing with 0.1M phosphate buffered saline (PBS, pH 7.4), the samples were incubated with Alexa Fluor-488-conjugated goat anti-rabbit IgG (1:150 dilution, catalog no. RBaf48801; Fcmacs, Nanjing, China) and Tritc-conjugated goat anti-mouse IgG (1:150 dilution; catalog no. BA 1089; Boster, Wuhan, China), Tritic-conjugated goat anti-rabbit IgG (1:150 dilution; catalog no. BA1090; Boster, Wuhan, China), and Alexa Fluor-488-conjugated goat anti-Mouse IgG (1:150 dilution, catalog no. BA1126; Boster, Wuhan, China) secondary bodies for 2 h at 37 °C. The nuclei were then counterstained with 4’,6-diamidini-2- phenylindole (DAPI) (catalog no. 13G04A76; Boster, Wuhan, China), after which glycerin was used for mounting. Using a BX53 Olympus microscope, sections were observed, and a digital color camera, namely, an Olympus DP73, was used for capturing fluorescent images.

## 5. Conclusions

Our results confirm the existence of TCs and long process Tps between lobules and gland cells within the connective tissue in the pancreas of Chinese soft-shelled turtles. TCs establish homo-cellular and hetero-cellular junctions that are involved in intercellular communications. TCs reduce the work load by releasing shading/secretary vesicles or exosomes for long and short distances. These conveys information to the damaged tissue for repair. Exosomes perform multifunction, specific immune reactions with macrophages or lymphocytes, peristaltic contraction in smooth muscles, chemical reactions in the case of damaged blood vessels, and repair. This information will help us to better understand the role of immune systems and different immunological disorders in the pancreas of reptiles.
